# Benzbromarone Attenuates Oxidative Stress in Angiotensin II- and Salt-Induced Hypertensive Model Rats

**DOI:** 10.1155/2018/7635274

**Published:** 2018-06-05

**Authors:** Nanako Muraya, Daisuke Kadowaki, Shigeyuki Miyamura, Kenichiro Kitamura, Kohei Uchimura, Yuki Narita, Yohei Miyamoto, Victor Tuan Giam Chuang, Kazuaki Taguchi, Toru Maruyama, Masaki Otagiri, Sumio Hirata

**Affiliations:** ^1^Department of Clinical Pharmacology, Kumamoto University, 5-1, Oe-Honmachi, Chuo-ku, Kumamoto 862-0973, Japan; ^2^Center for Clinical Pharmaceutical Sciences, Faculty of Pharmaceutical Sciences, Kumamoto University, 5-1, Oe-Honmachi, Chuo-ku, Kumamoto 862-0973, Japan; ^3^Department of Clinical Pharmaceutics, Faculty of Pharmaceutical Sciences, Sojo University, 4-22-1 Ikeda, Nishi-ku, Kumamoto 860-0082, Japan; ^4^DDS Research Institute, Sojo University, 4-22-1 Ikeda, Nishi-ku, Kumamoto 860-0082, Japan; ^5^Department of Biopharmaceutics, Graduate School of Pharmaceutical Sciences, Kumamoto University, Kumamoto, Japan; ^6^Third Department of Internal Medicine, Interdisciplinary Graduate School of Medicine and Engineering, University of Yamanashi, 1110 Shimokato, Chuo, Yamanashi 4093898, Japan; ^7^School of Pharmacy, Monash University Malaysia, Level 5, Building 2, Malaysia Campus, Jalan Lagoon Selatan, Bandar Sunway, 47500 Subang Jaya, Selangor, Malaysia; ^8^Department of Biopharmaceutics, Faculty of Pharmaceutical Sciences, Sojo University, Kumamoto, Japan

## Abstract

Oxidative stress induced by hyperuricemia is closely associated with the renin-angiotensin system, as well as the onset and progression of cardiovascular disease (CVD) and chronic kidney disease (CKD). It is therefore important to reduce oxidative stress to treat hyperuricemia. We previously found that benzbromarone, a uricosuric agent, has a direct free radical scavenging effect *in vitro*. The antioxidant effects of benzbromarone were evaluated *in vivo* via oral administration of benzbromarone for 4 weeks to model rats with angiotensin II- and salt-induced hypertension. Benzbromarone did not alter plasma uric acid levels or blood pressure but significantly reduced the levels of advanced oxidation protein products, which are oxidative stress markers. Furthermore, dihydroethidium staining of the kidney revealed a reduction in oxidative stress after benzbromarone administration. These results suggest that benzbromarone has a direct antioxidant effect *in vivo* and great potential to prevent CVD and CKD.

## 1. Introduction

The activation of the renin-angiotensin system (RAS) is closely related to the progression and development of cardiovascular disease (CVD) and chronic kidney disease (CKD) [1, 2]. It has been suggested that angiotensin II (ANG II) not only increases blood pressure by binding to angiotensin II type 1 (AT1) receptors but also produces reactive oxygen species (ROS) via the activation of NADPH oxidase [3, 4]. Excessive ROS promotes the vasoconstriction, proliferation, and hypertrophy of vascular smooth muscle cells, inducing endothelial cell dysfunction and inflammatory response in the vessel wall, which can cause heart or kidney dysfunction and failure [5].

Hyperuricemia has also been reported to be associated with CVD and CKD [6–8], in which vascular disorders mediated by oxidative stress have been reported [9]. In hyperuricemia, excess uric acid is taken up by vascular cells or adipocytes [10, 11]. The intracellular uric acid then activates NADPH oxidase, which produces ROS. Excess uric acid also causes a vicious cycle by activating local RAS, which further increases oxidative stress [12]. Thus, in order to prevent CVD and CKD, it is important to suppress the oxidative stress produced by uric acid.

Benzbromarone is a therapeutic agent that has been used clinically to combat hyperuricemia for more than 30 years. It facilitates the excretion of uric acid into urine by inhibiting proximal tubular uric acid transporter 1 (URAT1) [13]. We have previously shown that benzbromarone has a direct scavenging activity against superoxide radicals and reduces the levels of intracellular ROS produced by ANG II as well as uric acid in vascular endothelial cells [14]. Therefore, we predicted that benzbromarone has an antioxidant effect against URAT1-independent oxidative stress.

RAS activation has been reported to be involved in hyperuricemia-related organ damage [15–17]. In the present study, we evaluated the antioxidant activity of benzbromarone *in vivo* using a rat model of angiotensin II- and salt-induced hypertension. Benzbromarone was orally administered to the rats for 4 weeks, during which they were monitored for oxidative stress markers, blood pressure, and renal function. The results were then compared with those of model rats treated with olmesartan, an AT1 receptor blocker with antioxidant activity. These rats served as a positive control [18–20].

## 2. Materials and Methods

### 2.1. Materials

Chloramine-T was purchased from Nacalai Tesque Inc. (Kyoto, Japan). Methylcellulose 400, benzbromarone, dihydroethidium (DHE), and ANG II were purchased from Wako Pure Chemical Industries Ltd. (Osaka, Japan). Olmesartan was a kind gift from Daiichi Sankyo Pharmaceutical Co. Ltd. (Tokyo, Japan). All other chemicals were of the highest grade and obtained from commercial sources.

### 2.2. Animals

Six-week-old male Sprague-Dawley (SD) rats were purchased from Kyudo Co. Ltd. (Saga, Japan). The experimental protocol was reviewed and approved (F23-275) by the Animal Care and Use Committee of the School of Medicine, Kumamoto University. A notification was submitted to the Japanese government prior to commencement of the study. The rats used in the experiments were fed with ordinary laboratory chow, allowed free access to water, and maintained in a regular 12-hour light-dark cycle.

### 2.3. Preparation of ANG II-Salt-Infused Hypertension Model Rats

The hypertension model (ANG II-salt) rats were prepared by administering ANG II and NaCl to the rats according to a previously reported method [21, 22]. In brief, NaCl (1%) was given in the drinking water, and ANG II (120 ng/min) was subcutaneously infused using an implanted osmotic minipump (ALZET model 2004; Durect Corp., Cupertino, CA). The rats were randomly divided into 4 groups: (1) control rats, sham-operated; (2) ANG II-salt rats administered with vehicle; (3) ANG II-salt rats administered with benzbromarone (200 mg/kg per day); and (4) ANG II-salt rats administered with olmesartan (5 mg/kg per day). Vehicle, benzbromarone, and olmesartan were administered daily for 28 days through a stomach tube. Rodents generally have lower serum urate levels than humans due to the presence of uricase. Therefore, we administered a higher dose of benzbromarone based on a preclinical safety data by Urinorm®. Blood pressure was measured by the tail-cuff method using a BP-98E manometer (Muromachi Kikai, Osaka, Japan). In brief, conscious rats were placed in a restrainer on a warming pad and allowed to rest inside their cages before blood pressure was measured. Rat tails were placed inside a tail cuff, which was inflated and released several times to allow the animal to be conditioned for the procedure. Twenty-four-hour urine was collected from inside metabolic cages. Plasma was obtained by centrifugation of blood sample at 3000 rpm for 10 min and stored at −80°C until analysis. Blood pressure, blood sample, and urine sample were obtained at 0, 2, and 4 weeks. The survival rate of each group was monitored over the 4-week period.

### 2.4. Measurement of Physiologic Parameters

Creatinine (Cr) and blood urea nitrogen (BUN) were measured using LabAssay™ Creatinine (Wako Pure Chemical Industries Ltd., Osaka, Japan) and UNB-test Wako (Wako Pure Chemical Industries Ltd., Osaka, Japan), respectively. Total protein (TP) and urinary protein (U-pro) were determined using the Bradford method.

Uric acid (UA), aspartate aminotransferase (AST), and alanine aminotransferase (ALT) were measured using LabAssay Uric Acid (Wako Pure Chemical Industries Ltd., Osaka, Japan) and transaminase CII-test Wako (Wako Pure Chemical Industries Ltd., Osaka, Japan), respectively. All procedures were performed according to the manufacturer's instructions.

### 2.5. Kidney Histopathology

Harvested kidney tissues were fixed in 4% paraformaldehyde. The tissues were embedded in paraffin blocks and sectioned at 2 *μ*m thickness for histologic examination. Paraffin sections of kidney tissue were stained with periodic acid-Schiff (PAS), hematoxylin and eosin (H&E), or Azan-Mallory stain.

### 2.6. Measurement of Oxidative Stress Markers

Advanced oxidation protein products (AOPPs) were measured by using a previously reported method [23]. Plasma samples were diluted 10 times with phosphate-buffered saline (PBS). To the diluted plasma sample (200 *μ*L), 10 *μ*L 1.16 M potassium iodide (KI) and 20 *μ*L acetic acid were added. After incubation for 30 min, the absorbance was measured at 340 nm using a fluorescence microplate reader (Spectra Fluor, Tecan Group Ltd., Männedorf, Switzerland). The AOPP concentration was calculated from a standard curve using chloramine-T.

### 2.7. Detection of Reactive Oxygen Species (ROS) in the Kidney Tissue

The fluorescent dye DHE was used to detect ROS in the kidney as previously described [24]. Seven-micrometer cryosections of kidney tissues were stained with the superoxide-sensitive dye DHE (100 *μ*mol/L) in a light-protected and humidified chamber for 30 min at 37°C. The results obtained from three experiments were quantified as fluorescence intensity.

### 2.8. Statistical Analysis

The results are reported as the mean ± SD. Statistical significance was evaluated using analysis of variance (ANOVA), followed by the Tukey-Kramer post hoc test. For all analyses, *P* < 0.05 was regarded as statistically significant.

## 3. Results

### 3.1. Changes in Biochemical Parameters and Blood Pressure after the Administration of Benzbromarone

**Figure 1:**
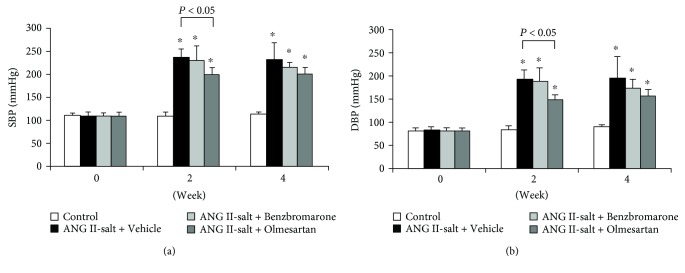
Benzbromarone had no effect on SBP (a) and DBP (b) in ANG II/salt-treated rats. Benzbromarone (200 mg/kg per day), olmesartan (5 mg/kg per day), or vehicle was administered once daily for 28 days through a stomach tube. Values are expressed as the mean ± SD (control, *n* = 5; others, *n* = 4–11). ^∗^*P* < 0.05 compared with the control group.

At 2 weeks after the administration of benzbromarone, blood pressure in the ANG II- and salt-treated group was higher than that in the control group. Neither benzbromarone nor vehicle treatment affected systolic blood pressure (SBP) and diastolic blood pressure (DBP). In contrast, the olmesartan-treated group showed significantly reduced SBP (Figure 1(a)) and DBP (Figure 1(b)) at 2 weeks, which was sustained up to 4 weeks.

Table 1 shows the changes in biochemical parameters. Although an increase in urinary protein and blood urea nitrogen (BUN) was observed in the benzbromarone and vehicle groups, there was no change in creatinine. Therefore, benzbromarone had no effect on renal function (Table 1). In contrast, the olmesartan group showed suppressed urinary protein and BUN. These changes in markers of renal function depended mainly on the action of ANG II. The AST and ALT values of the benzbromarone-treated group at week 4 were slightly increased compared to those at week 0, but the values were within the normal range found in humans, which are almost the same as those in rodents. Therefore, benzbromarone did not induce significant liver damage. At week 4, the uric acid level was slightly increased in the vehicle-treated group, despite remaining low in all other groups including the benzbromarone group. Hence, the effect of the various treatments on uric acid was insignificant.

### 3.2. Effect of Benzbromarone on Oxidative Stress

**Figure 2:**
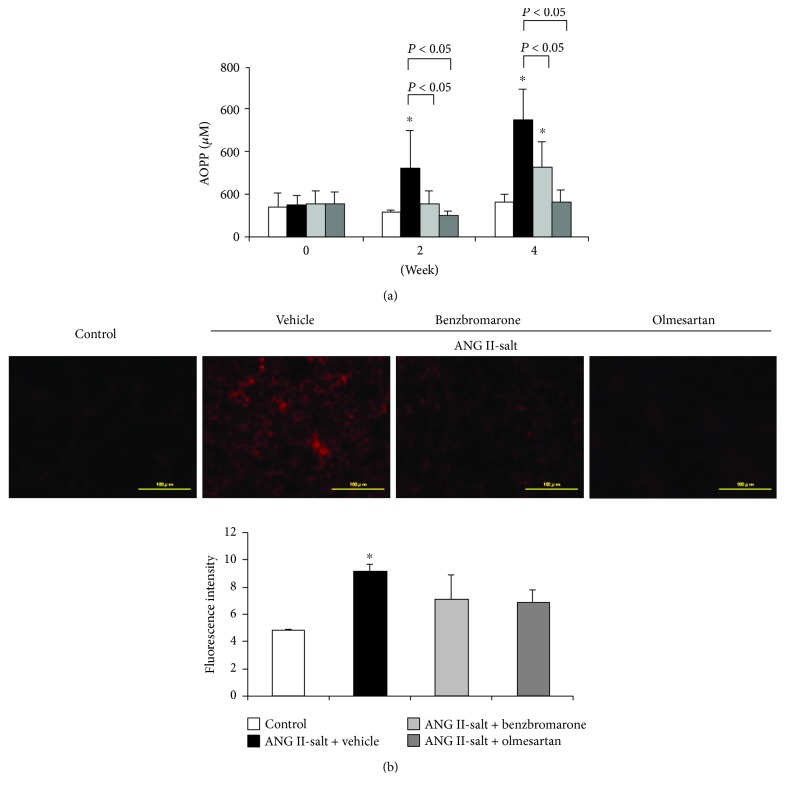
Effects of benzbromarone on markers of oxidative stress. Changes in serum-advanced oxidation protein product (AOPP) levels (a) and dihydroethidium (DHE) staining (b) of frozen kidney sections. Values are expressed as the mean ± SD (a) (control, *n* = 5; others, *n* = 4–11), (b) magnification: ×40. Scale bars: 100 *μ*m. ^∗^*P* < 0.05 compared with the control group.

The vehicle-treated group showed a significant increase in plasma concentrations of advanced oxidation protein products (AOPPs) after 2 weeks, in contrast to the olmesartan- and benzbromarone-treated groups, which showed a significant decrease in AOPPs (Figure 2(a)). In addition, DHE staining of the renal tissues showed increased fluorescence intensity in the vehicle-treated group and decreased intensity in both olmesartan- and benzbromarone-treated groups (Figure 2(b)).

### 3.3. Morphologic Changes in Kidney Tissue after Administration of Benzbromarone

**Figure 3:**
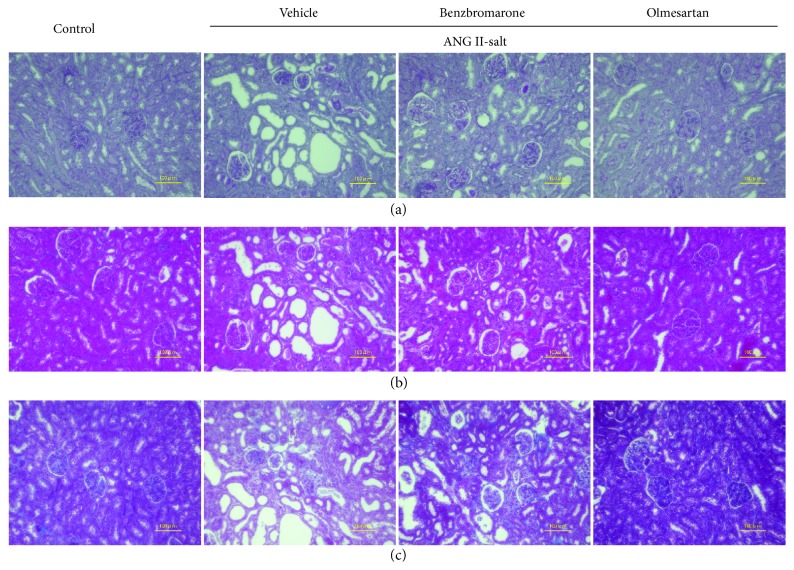
Morphological changes in kidney tissues after administration of benzbromarone. Paraffin sections of the kidney tissue were stained with periodic acid-Schiff (PAS) stain (a), hematoxylin and eosin (H&E) (b), and Azan-Mallory stain (c). Magnification: ×200. Scale bars: 100 *μ*m.

Kidney tissue was subjected to PAS and H&E staining for the examination of morphological changes 4 weeks after benzbromarone administration. Tubular dilation was observed in the vehicle-treated group but not in the benzbromarone-treated group, indicating that benzbromarone tends to suppress this damage (Figures 3(a) and 3(b)). Mesangial cell proliferation was not observed in all groups, whereas interstitial fibrosis was observed in all groups, as revealed by Azan-Mallory staining (Figure 3(c)).

## 4. Discussion

After 2 weeks of administration of ANG II and salt, high blood pressure, increased urinary protein excretion, and increased plasma levels of AOPP, an oxidative stress marker, were observed in the vehicle-treated group (Figure 1, Table 1). In contrast, benzbromarone treatment reduced the levels of AOPP, suggesting that benzbromarone may exhibit antioxidant effects *in vivo* (Figure 2). Morphological examination of kidney tissues (Figure 3) indicated that the benzbromarone-treated group showed a tendency to suppress tubular dilation, in contrast to the vehicle-treated group. The suppression of DHE-stained cells in the benzbromarone group suggests that the improvement in oxidative stress status may have a protective effect on organs such as the kidneys. A possible mechanism of benzbromarone-induced reduction in oxidative stress is through the activation of RAS or inhibition of URAT1. However, our results clearly showed that it was olmesartan that exerted antihypertensive effects and reduced urinary protein excretion. Thus, benzbromarone had no effect on RAS activation in this experiment. Furthermore, the serum uric acid levels observed were lower in ANG-II-infused hypertensive model rats than in normal rats, suggesting that the URAT1-inhibitory effect of benzbromarone may not be responsible for the reduction in oxidative stress. Rodents generally show a lower serum urate level than humans due to the presence of uricase, which converts urate to allantoin [15]. Taken together, the antioxidant effect of benzbromarone was independent of its inhibitory effect on RAS and URAT1.

Benzbromarone inhibits uric acid-induced oxidative stress by inhibiting the uptake of uric acid into the vascular endothelial or smooth muscle cells [11]. In addition, benzbromarone has been reported to partially inhibit voltage-driven urate transporter 1 (URATv1), the extracellular uric acid efflux transporter, in renal tubular cells [25]. Interestingly, benzbromarone reduced both urate-dependent ROS [26] and NO [27] levels by inhibiting URATv1. URAT1 and URATv1 exist in various tissues, including vascular smooth muscle and endothelial cells. Further studies are necessary to clarify whether benzbromarone might affect both URAT1 and URATv1 in CKD or CVD. Although inhibitors of uric acid production, such as allopurinol, also have antioxidant effects owing to their xanthine oxidase-inhibitory action [28], benzbromarone is considered superior because it can trap ROS derived from both xanthine oxidase and NADPH oxidase. In fact, recent clinical reports on patients with hyperuricemia or heart failure suggest that benzbromarone improves the function of endothelial cells in terms of flow-mediated dilation (FMD) and insulin resistance, resulting in effective organ protection [29, 30].

To the best of our knowledge, our study is the first to report that benzbromarone exerts direct antioxidant effects *in vivo*. It has been shown that intracellular RAS is activated by ROS derived from uric acid [12] and the direct antioxidant effect of benzbromarone may contribute to inhibiting local RAS activation in hyperuricemia. On the contrary, some studies have shown that hyperuricemic patients commonly have hypertension [31, 32] due to systemic RAS activation following the elevation in uric acid levels [15–17]. RAS-induced oxidative stress is closely related to organ damage [5]; thus, benzbromarone, in directly reducing oxidative stress due to RAS, may be a valuable clinical agent that can be used for organ protection. Based on the drug information for benzbromarone (Urinorm), the dose of benzbromarone was comparatively high in this *in vivo* study; therefore, further clinical studies will be required to clarify the antioxidant activity of benzbromarone. Additionally, because benzbromarone slightly increases serum ALT levels, its safety also requires clinical evaluation. We speculate that as serum urate levels are higher in humans than in rodents, benzbromarone might act as an antioxidant at lower concentrations.

On the contrary, olmesartan, an AT1 receptor blocker (ARB), was also shown to have high antioxidant activity, with effects similar to those reported previously [18–20] in model rats with ANG-II-induced hypertension. However, ARBs can elevate serum uric acid levels [33]. Therefore, the serum uric acid levels of CKD patients receiving ARBs are normally monitored. From the perspective of achieving synergistic treatment effects from antioxidants and antihyperuricemics, the combination therapy of olmesartan and benzbromarone may be a better option for CKD patients with hyperuricemia.

## 5. Conclusion

In this study, we showed that benzbromarone exerted antioxidant activity, independent of its blockade of RAS or URAT1, against AII-induced ROS *in vivo*. Our results suggest that benzbromarone may reduce not only uric acid-dependent oxidative stress induced by the intracellular uptake of uric acid via URAT1 but also nonuric acid-dependent ROS, such as those derived from NADPH oxidase activated by ANG II. Benzbromarone may therefore be useful as an antioxidant for the prevention of CVD and CKD.

## Figures and Tables

**Table 1:** Physiological parameters at 0, 2, and 4 weeks.

Week	Control			ANG II-salt								
				Vehicle			Benzbromarone			Olmesartan		
	0	2	4	0	2	4	0	2	4	0	2	4
Body weight (g)	209 ± 13	348 ± 16	405 ± 41	205 ± 13	204 ± 33^∗^	197 ± 42^∗^	205 ± 12	239 ± 31^∗^	224 ± 50^∗^	208 ± 14	304 ± 14^∗^^,#^	363 ± 21^#^
TP (g/dL)	5.7 ± 0.2	5.8 ± 0.1	6.0 ± 0.2	5.4 ± 0.2	5.7 ± 0.6	5.9 ± 0.5	5.4 ± 0.3	5.5 ± 0.6	6.1 ± 0.8	5.6 ± 0.1	6.0 ± 0.4	6.4 ± 0.4
UA (mg/dL)	0.48 ± 0.14	0.78 ± 0.34	0.68 ± 0.19	0.49 ± 0.22	0.63 ± 0.59	1.77 ± 1.22^∗^	0.53 ± 0.27	0.89 ± 0.43	1.04 ± 0.29	0.40 ± 0.14	0.92 ± 0.44	0.56 ± 0.34^#^
Cr (mg/dL)	0.54 ± 0.12	0.79 ± 0.17	0.45 ± 0.12	0.50 ± 0.23	0.74 ± 0.23	0.84 ± 0.48	0.61 ± 0.18	0.47 ± 0.35	0.95 ± 0.69	0.48 ± 0.07	0.87 ± 0.59	0.61 ± 0.37
BUN (mg/dL)	22.1 ± 10.9	16.5 ± 3.0	22.1 ± 3.4	22.6 ± 10.2	35.5 ± 10.2^∗^	42.0 ± 33.8	20.4 ± 6.9	39.6 ± 6.3^∗^	42.2 ± 21.1	18.1 ± 7.4	21.5 ± 4.8^#^	24.5 ± 6.3
AST (IU/L)	28.8 ± 1.8	30.6 ± 4.9	28.8 ± 3.6	31.2 ± 3.8	29.5 ± 2.5	31.6 ± 10.2	30.9 ± 3.6	34.8 ± 12.1	42.0 ± 19.6	29.6 ± 4.0	32.9 ± 3.3	27.5 ± 2.2
ALT (IU/L)	7.5 ± 1.5	7.6 ± 1.0	8.2 ± 1.1	7.7 ± 0.9	9.7 ± 2.7	18.6 ± 5.6^∗^	7.8 ± 1.1	11.1 ± 3.3	16.5 ± 7.2^∗^	7.1 ± 1.4	9.3 ± 1.4	9.4 ± 2.1^#^
U-pro (mg/day)	4 ± 2	8 ± 2	9 ± 2	4 ± 2	313 ± 170^∗^	255 ± 278^∗^	4 ± 3	255 ± 100^∗^	255 ± 88^∗^	5 ± 3	14 ± 8^#^	18 ± 11^#^

Values are expressed as the means ± SD (control, *n* = 5, others, *n* = 3–11). ^∗^*P* < 0.05 compared with the control group, ^#^*P* < 0.05 compared with the vehicle group at the same week.

## Data Availability

The data used to support the findings of this study are available from the corresponding author upon request.
